# The clinical course and outcomes of non-aneurysmal subarachnoid hemorrhages in a single-center retrospective study

**DOI:** 10.1007/s00701-023-05767-4

**Published:** 2023-09-02

**Authors:** Jeremias Tarkiainen, Valtteri Hovi, Liisa Pyysalo, Antti Ronkainen, Juhana Frösen

**Affiliations:** 1 Department of Neurosurgery, Tampere University Hospital and University of Tampere, Tampere, Finland; 2 Hemorrhagic Brain Pathology Research Group, Faculty of Medical Technology and Health Sciences, Tampere University, Tampere, Finland; 3 Department of Rehabilitation, Tampere University Hospital, Tampere, Finland

**Keywords:** Non-aneurysmal subarachnoid hemorrhage, Loss of consciousness, Hydrocephalus, Vasospasm, Outcome

## Abstract

**Background:**

Non-aneurysmal subarachnoid hemorrhages (SAHs) are thought to have a benign clinical course compared to aneurysmal SAHs. The aim of this study is to report the clinical course and outcomes of non-aneurysmal SAHs in a large single-center study.

**Methods:**

The patients with non-aneurysmal SAHs were screened from Tampere University Hospital from 2005 to 2020. The clinical data were collected from the patient’s medical records and from the imaging studies. The primary interest was the neurological outcome assessed by dichotomized GOS at 2 months. Multivariable logistic regression was used to study the factors associated with unfavorable outcome.

**Results:**

We found 216 non-aneurysmal SAHs in 214 patients (2 patients with > 1 bleed). Ninety-seven percent of patients with a typical perimesencephalic bleeding pattern SAH (PSAH) (75/77) had a favorable outcome, while 86% of patients with non-perimesencephalic SAH (NPSAH) had a favorable outcome (84/98). In a multivariable logistic regression analysis, loss of consciousness (LOC) (aOR 214.67, 95% CI 17.62–2615.89) and Fisher grade 4 bleeding pattern (aOR 23.32, 95% CI 1.40–387.98) were associated with increased risk for unfavorable outcome (GOS 1–3). Vasospasm was seen in 20% of non-aneurysmal SAH patients, hydrocephalus in 17%, and 13% needed ventriculostomy.

**Conclusions:**

Non-aneurysmal SAH seems to have a good prognosis for majority of patients, especially for patients with a PSAH. Non-aneurysmal SAH patients are however affected by vasospasm and hydrocephalus and have similar risk factors for poor outcome as patients with aneurysmal SAH. This suggests that it is the severity of the bleed rather than the etiology that associates with poor outcome.

**Supplementary Information:**

The online version contains supplementary material available at 10.1007/s00701-023-05767-4.

## Introduction

**Fig. 1:**
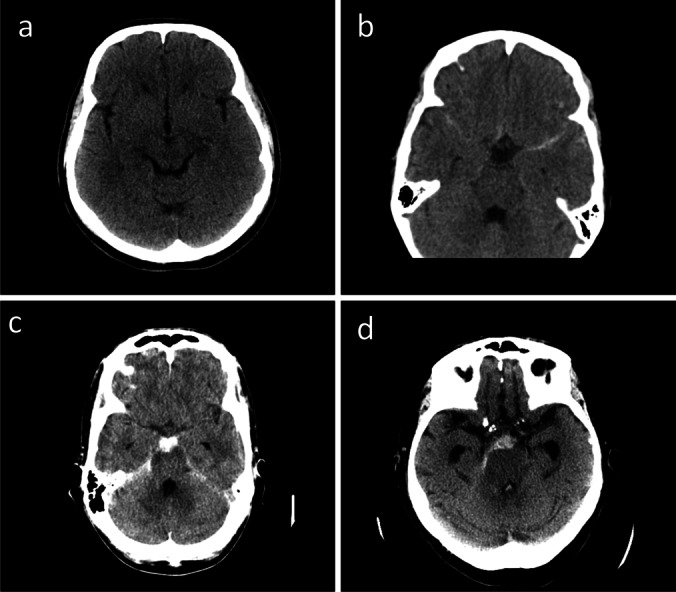
Non-aneurysmal SAHs with an unknown etiology. **a** Fisher grade 1 SAH. The diagnosis of SAH is based on lumbar puncture and **b** Fisher grade 2 SAH. **c** A typical non-aneurysmal SAH Fisher grade 3 SAH. **d** Fisher grade 4 non-aneurysmal SAH, the blood is also present in the fourth ventricle

Spontaneous subarachnoid hemorrhage (SAH) accounts for 5% of strokes [5]. Unlike other types of strokes, SAH affects mainly working aged patients as the median age for SAH is around 55 years [20]. Thus, despite being a small proportion of strokes (5%) SAHs account for approximately 27% of all stroke-related life years lost [8]. Around 85% of SAH cases are related to the bleeding of an intracranial aneurysm. However, of the 15% SAH cases that are not related to aneurysms, the etiology for bleeding remains unknown for 10%. The remaining 5% are related to various rare conditions such as cerebral AVMs, arterial dissections, or dural arteriovenous fistulae [21]. The bleedings with an unknown etiology are usually classified as non-aneurysmal spontaneous subarachnoid hemorrhages (SAH) (Fig. 1), which can further be divided into perimesencephalic SAH (PSAH), in which the blood is centered anterior to the midbrain/pons area, and non-perimesencephalic SAH (NPSAH), in which the bleeding pattern is more diffuse [15].

It is thought that non-aneurysmal SAHs have a benign clinical course compared to aneurysmal SAHs (aSAH). Schuss et al. [16] studied outcomes of non-aneurysmal SAHs. They concluded that 87% of non-aneurysmal SAH patients recover with favorable outcome (mRS 0–2) [16]. However, they also reported that shunt dependency develops for 14% of patients, and the risk for shunt dependency is especially higher in patients with NPSAH (20% vs 5%). A similar study was conducted in Finland by Achrén et al. [1] where they reported similar findings as 84% recovered with favorable outcome (GOS 4–5) and 17% of patients developed shunt dependency, suggesting that non-aneurysmal SAH patients also suffer from chronic post SAH complications despite the favorable outcome. As in the study by Schuss et al. [16], they found that patients with NPSAH were in increased risk to develop hydrocephalus and shunt dependency compared to patients with PSAH [1].

The aim of this study is to determine the clinical course and outcomes of non-aneurysmal SAH patients in a large single-center cohort study.

## Material and methods

**Fig. 2:**
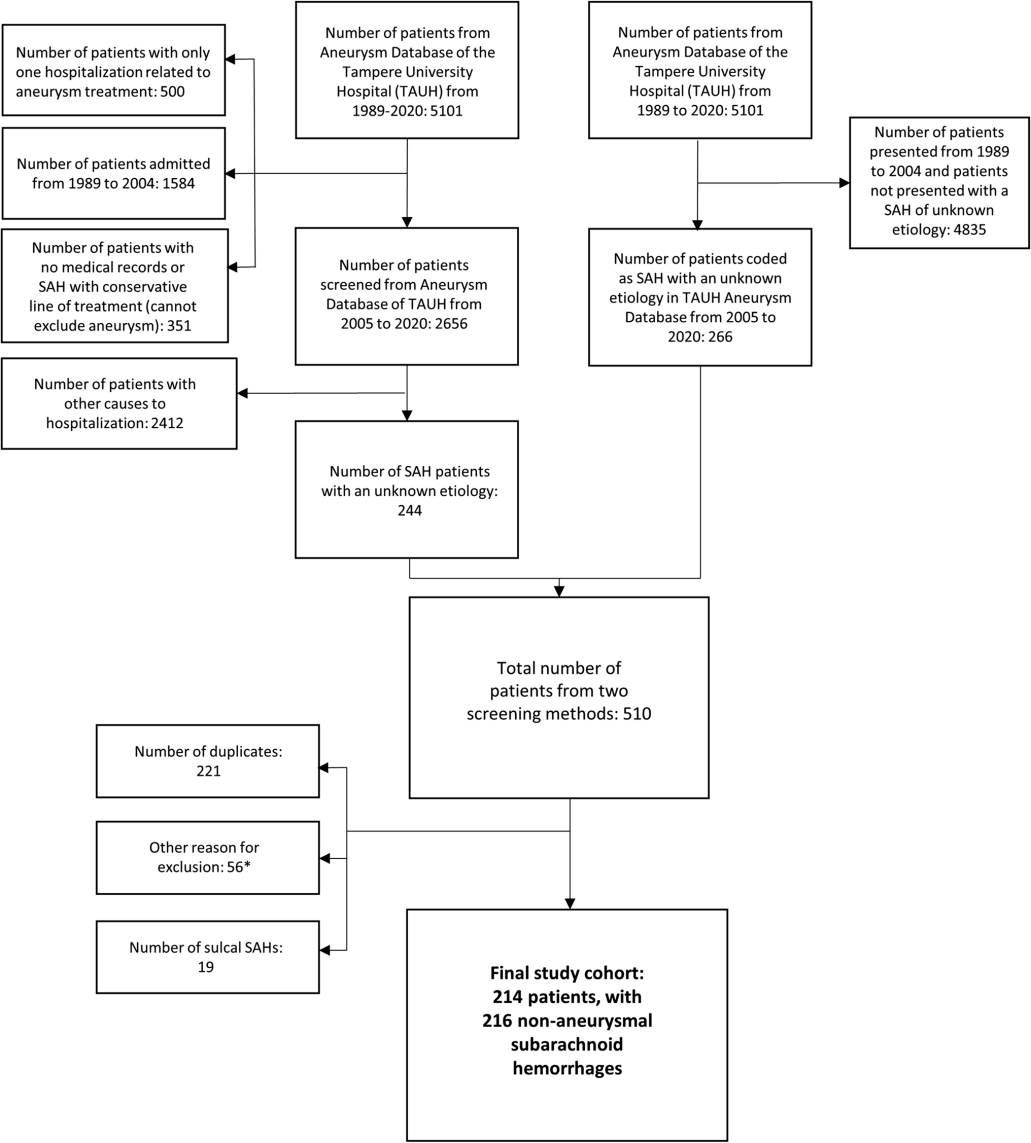
A flowchart describing how the study cohort was formed. The cohort was formed by two screening methods in order to include all cases. As the two screening methods produced similar data, it also validates the comprehensiveness of the TAUH Aneurysm Database. *The group of patients with other reason for exclusion included patients of whom medical records were not available, patients with only native CT or patients of whom the bleeding source were eventually found, such as blister aneurysm or moyamoya disease

Tampere University Hospital (TAUH) is the only neurosurgical unit providing treatment for SAHs in its catchment area, making it the primary referral center for patients presented with SAH. The TAUH aneurysm database is a single institution database into which all patients presented with SAH (ICD I60.1–9) or unruptured aneurysms (ICD I67.1) in its catchment area are entered from 1989 onwards. All patients with a potential non-aneurysmal SAH were identified and screened from 2005 to 2020 from the TAUH Aneurysm Database (Fig. 2). TAUH implemented the use of digital image archive in 2003–2004. Since 2005, the imaging studies and medical patient’s records are available constantly and therefore that was chosen as the starting point for this study. As the study is retrospective and the data was collected from the Aneurysm Database and from the patient’s medical records or the digitally archived imaging studies, the TAUH ethics committee waived the need for informed consent of the participating patients. This study was conducted according to the STROBE reporting guidelines (Data supplement).

In accordance with the European Union’s GDPR regulations, the data used in this study cannot be made freely available. Pseudonymized dataset can, however, be shared after formal approval of a scientific research plan and data management plan by the local ethical review board and Tampere University Hospital.

### Data collection

The data was collected from the TAUH Aneurysm Database. In addition, the imaging studies and patient’s medical records were reviewed by a single author to ensure consistency of the data. Bleeding pattern, vasospasm, and the clinical and treatment-related data, such as age, sex, initial loss of consciousness (LOC), Glasgow Coma Scale (GCS) on site and on admission, World Federation of Neurosurgical Societies (WFNS) grade, Glasgow Outcome Scale (GOS) at 2 months, hydrocephalus, or requirement of ventriculostomy or shunt were reviewed. GCS change was defined as GCS on site minus GCS on admission to the hospital. The distribution of bleed (PSAH vs NPSAH) was reviewed from the imaging studies. PSAH was defined as follows: (1) the blood is centered anterior to midbrain/pons area, (2) the bleed may extend into basal and suprasellar cisterns and into the proximal part of the Sylvian fissure, and (3) no overt intraventricular extension. Presence of hydrocephalus was determined according to the diagnosis made by the treating physicians on the medical records. Vasospasm was defined as radiological vasospasm detected in imaging studies. The primary interest was the neurological outcome. GOS was determined according to the latest neurological outcome of the patient recorded in the patient records by 2 months after the SAH. Follow-up visits were not routinely scheduled for some of the patients with mild non-aneurysmal SAHs that were discharged in good condition (46% of GOS 5 classified patients). For them, GOS at 2 months was categorized as 5 unless recorded otherwise in the patient records. Fifty-four percent (99/183) of GOS 5 classified patients had a scheduled follow-up. For those patients discharged with disability (GOS 4–2), the GOS was determined according to the neurological status at the latest follow-up visit by 2 months. Patients with uncertain outcome at 2 months were excluded from the analysis.

### Statistical analysis

Proportions and percentages were calculated for categorical variables and compared with chi-square test between groups. Medians and ranges were calculated for non-normally distributed continuous variables and compared between groups using the Mann–Whitney *U* test. Univariate logistic regression analysis was used to analyze the impact of patient and bleeding-related factors on the neurological outcome (dichotomized GOS), by calculating ORs and its corresponding 95% confidence intervals (CIs). In addition, a multivariate logistic regression model was used to identify factors independently associated with SAH outcome. Results from those analyses were reported as adjusted ORs (aORs) with 95% CIs. Significant variables associated with unfavorable outcome were included in the multivariate analysis. WFNS grade and the need for ventriculostomy were not included in the model to avoid multicollinearity with LOC and hydrocephalus. The data was analyzed using SPSS version 26.0 statistical software (IBM, Armonk, USA).

## Results

**Fig. 3:**
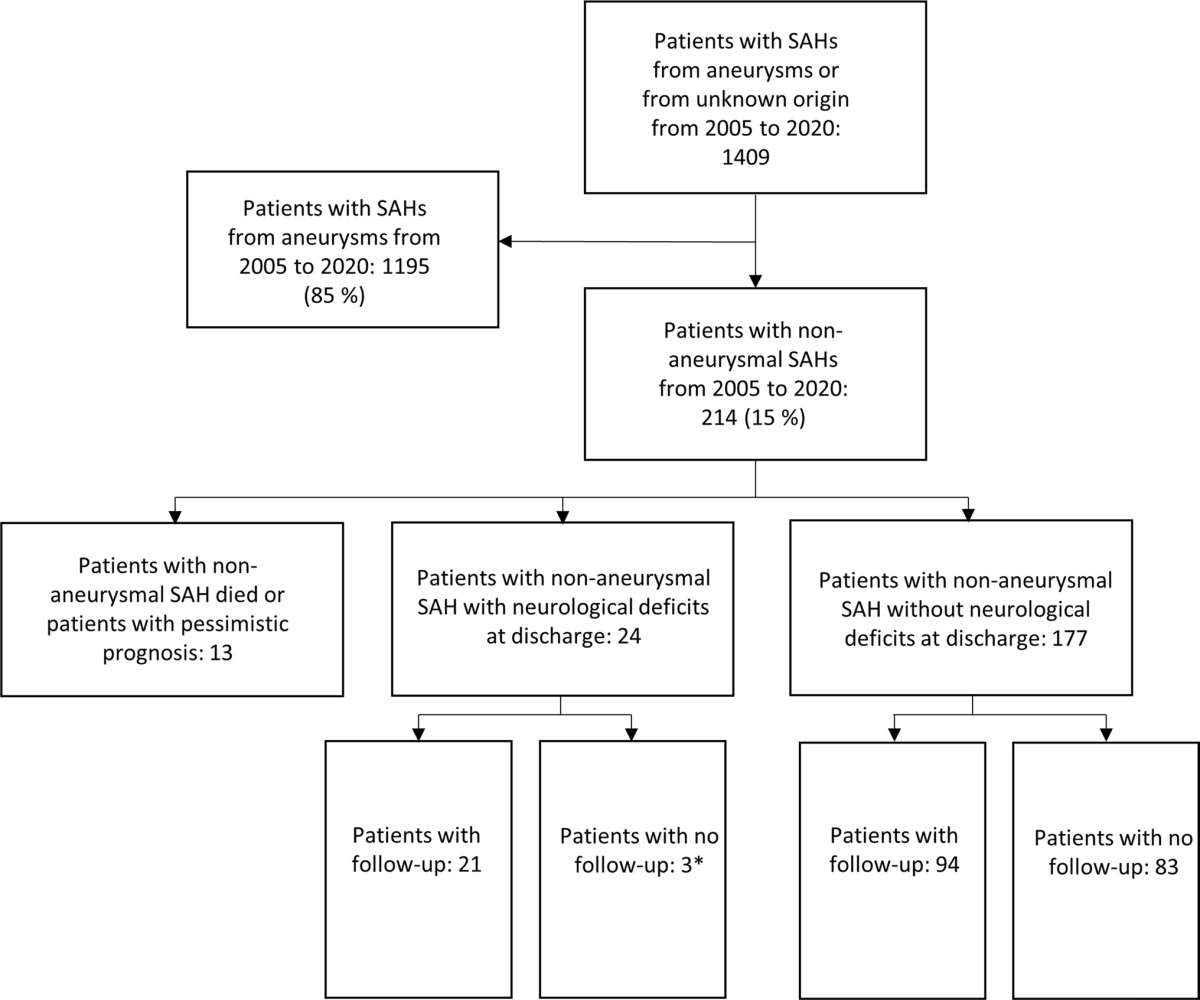
A flowchart describing how the follow-up was implemented. *Out of 3 patients with neurological deficits at discharge, one of them was included in the analysis of outcome due to only minor neurological deficits at discharge (GOS 5). Two patients were excluded from the outcome analysis as they were lost during follow-up with an uncertain condition

The screening process yielded 233 patients with 235 angiogram-negative SAHs (Fig. 2). When further analyzing the bleeding patterns of non-aneurysmal SAHs, 19/235 (8%) non-aneurysmal SAHs turned out to be sulcal SAHs, with a probable etiology of small vessel disease (SVD). These patients were excluded resulting in a total of 216 non-aneurysmal SAHs in 214 patients (two re-bleeds, incidence of re-bleed 0.9%). The GOS at 2 months could be determined for 128 patients. Eighty-four patients left the hospital with a good neurological state, and thus, routine follow-up was not scheduled for these patients. Final analysis of outcome included 212 patients. Two patients were excluded as they did not have follow-up and they left hospital with an uncertain neurological condition (Fig. 3).

### Patient characteristics of non-aneurysmal SAH patients and characteristics according to outcome

The median age for bleeding was 55 years and 51% were female (Table 1). Most of the patients had a GCS of 15 on admission to hospital, and the mean of GCS change (on site-on admission) was 0.2. Fisher grade 3 was the most common type of bleeding pattern (51%). A total of 16 patients (7.5%) had an unfavorable outcome (GOS 1–3). The median time for GOS assessment was 66 days (IQR 41–114). For patients with no follow-up due to good neurological state, the median time from bleeding to last day at hospital was 8 days. Eighty-one percent of patients with an unfavorable outcome had a LOC while only 3% of patients with favorable outcome had LOC. Low GCS score, high WFNS grade bleed, LOC, hydrocephalus, ventriculostomy, and Fisher grade were significantly associated with unfavorable outcome. The positive predictive value (PPV) for unfavorable outcome was 68% (13/19) for LOC and 9% (16/170) for Fisher grades 3 and 4. Almost all the bleeding patterns of unfavorable outcome patients were Fisher grade 4 (14/16). Of Fisher grades 1 and 2, none of the patients had an unfavorable outcome. Positive predictive value for favorable outcome was 100% (42/42) for Fisher grade 1 and 2 bleeding patters.

**Table 1:** The characteristics of all non-aneurysmal SAH patients and characteristics of non-aneurysmal SAHs with favorable and unfavorable outcomes

	All non-aneurysmal SAH patients (*N* = 214)	Favorable outcome (*N* = 196)	Unfavorable outcome (*N* = 16)	*P* value
Age, median (range)	55 (14–88)	55 (14–88)	59.5 (38–85)	0.215
Female	50.9% (109/214)	51.9% (100/196)	56.3% (9/16)	0.687
Loss of consciousness	8.9% (19/214)	3.1% (6/196)	81.3% (13/16)	< 0.001
GCS score				
15	81.8% (175/214)	88.3% (173/196)	6.3% (1/16)	< 0.001
13–14	8.9% (19/214)	9.2% (18/196)	6.3% (1/16)	
7–12	4.2% (9/214)	2.0% (4/196)	25.0% (4/16)	
< 7	5.1% (11/214)	0.5% (1/196)	62.5% (10/16)	
Change in GCS (mean)	0.20	0.15	0.69	0.058
WFNS 4–5	9.3% (20/214)	2.6% (5/196)	87.5% (14/16)	< 0.001
WFNS 1–3	90.7% (194/214)	97.4% (191/196)	12.5% (2/16)	< 0.001
WFNS 1–3 but also LOC	2.8% (6/214)	2.6% (5/196)	6.3% (1/16)	0.391
Fisher grade				
1	17.3% (37/214)	18.9% (37/196)	0.0% (0/16)	< 0.001
2	2.3% (5/214)	2.6% (5/196)	0.0% (0/16)	
3	50.5% (108/214)	53.6% (105/196)	12.5% (2/16)	
4	29.9% (64/214)	25.0% (49/196)	87.5% (14/16)	
Vasospasm	20.1% (43/214)	18.9% (37/196)	37.5% (6/16)	0.075
Hydrocephalus	16.8% (36/214)	11.7% (23/196)	75.0% (12/16)	< 0.001
Ventriculostomy	12.6% (27/214)	9.2% (18/196)	56.3% (9/16)	< 0.001
Spinal drainage	1.9% (4/214)	1.5% (3/196)	0.0% (0/16)	0.618
CSF shunt	3.7% (8/214)	3.6% (7/196)	6.3% (1/16)	0.589

### Patient characteristics according to the bleeding pattern

Forty-four percent (77/177) of patients with visible SAH had a typical PSAH bleeding pattern, while 56% of patients had more diffuse NPSAH bleeding pattern (Table 2). Seventeen percent of patients had a Fisher grade 1 SAH, with no visible blood in the subarachnoid space in a CT study. Thus, they were excluded from the analysis between PSAH and NPSAH. Patients with NPSAH had more frequently unfavorable outcome (14% vs 3%). The patients with NPSAH were older (57 vs 51 years old), had more frequently LOC at onset (14% vs 3%), had lower GCS score on admission, and had more often high WFNS and Fisher grade bleeds. The patients with NPSAH also developed more frequently hydrocephalus (30% vs 8%) and the need for ventriculostomy (22% vs 7%).

**Table 2:** Patient characteristics and treatment-related variables for patients with perimesencephalic SAH (PSAH) and patients with diffuse pattern non-perimesencephalic SAH (NPSAH)

	All PSAHs (*N* = 77)	All NPSAHs (*N* = 100)	*P* value
Age, median (range)	51 (28–85)	57 (20–88)	0.020
Female	54.5% (42/77)	48.0% (48/100)	0.388
Loss of consciousness	2.6% (2/77)	14.0% (14/100)	0.009
Favorable outcome	97.4% (75/77)	85.7% (84/98)	0.008
Re-bleed	0.0% (0/77)	2.0% (2/100)	0.212
GCS score			
15	90.9% (70/77)	69.0% (69/100)	< 0.001
13–14	7.8% (6/77)	13.0% (13/100)	
7–12	0.0% (0/77)	8.0% (8/100)	
< 7	1.3% (1/77)	10.0% (10/100)	
Change in GCS (mean)	0.29	0.18	0.424
WFNS 4–5	1.3% (1/77)	18.0% (18/100)	< 0.001
Fisher grade			
1	–	–	
2	2.6% (2/77)	3.0% (3/100)	< 0.001
3	76.6% (59/77)	49.0% (49/100)	
4	20.8% (16/77)	48.0% (48/100)	
Vasospasm	16.9% (13/77)	27.0% (27/100)	0.111
Hydrocephalus	7.8% (6/77)	30.0% (30/100)	< 0.001
Ventriculostomy	6.5% (5/77)	22.0% (22/100)	0.004
Spinal drainage	0.0% (0/77)	4.0% (4/100)	0.076
CSF shunt	2.6% (2/77)	6.0% (6/100)	0.280

### Performed angiograms

A total of 649 angiograms was performed on 214 patients. Repeated angiograms were performed to exclude aneurysms or other sources of bleeding. Of all angiograms, 37.3% were CTA, 45.0% DSA, and 17.7% MRA. The median number of angiograms per patient was 3 (IQR 2–4). 93.5% of patients had at least 2 angiograms and 71.5% of patients had 3 or more angiograms. 89.7% of patients underwent at least one DSA study, and 49.1% of patients underwent at least one MRA study.

### Impact of vasospasm and ventriculostomy-dependent hydrocephalus on the neurological outcome of non-aneurysmatic SAH patients

We found vasospasm in 20% of patients (43/214) and the need for ventriculostomy in 13% of patients (27/214) (Table 3). Both groups had worse outcomes compared to the patients without vasospasm or the need for ventriculostomy. Fourteen percent of patients with vasospasm and 33.3% of ventriculostomy patients had an unfavorable outcome compared to the reference groups (5.9 and 3.8%). In addition, these patient groups had more frequent LOC, 14% vs 8% (*P* = 0.186) for vasospasm patients and 30% vs 6% (*P* =  < 0.001) for patients with the need for ventriculostomy.

**Table 3:** The impact of vasospasm and ventriculostomy on neurological outcome

	No vasospasm (*N* = 171)	Vasospasm (*N* = 43)	No ventriculostomy (*N* = 187)	Ventriculostomy (*N* = 27)
Poor neurological outcome (GOS 1–3 at 2 months)	5.9% (10/169)	14.0% (6/43)	3.8% (7/185)	33.3% (9/27)
WFNS grade of 4–5 on admission	6.4% (11/171)	20.9% (9/43)	4.3% (8/187)	44.4% (12/27)
WFNS grade of 1–3 on admission	93.5% (160/171)	79.1% (34/43)	95.7% (179/187)	55.6% (15/27)
GCS 15 on admission	82.3% (144/171)	72.1% (31/43)	88.2% (165/187)	37.0% (10/27)
Loss of consciousness	7.6% (13/171)	14.0% (6/43)	5.9% (11/187)	29.6% (8/27)

### Logistic regression analysis

A total of 212 patients were included in the logistic regression analysis (Table 4) to analyze predictors of poor functional outcome (GOS 1–3). In a univariate analysis, LOC (OR 137.22, 95% CI 30.76–612.21), WFNS grade of 4–5 (OR 267.40, 95% CI 47.54–1504.23), Fisher grade 4 (OR 21.00, 95% CI 4.61–95.68), hydrocephalus (OR 22.57, 95% CI 6.71–75.85), and ventriculostomy (OR 12.71, 95% CI 4.23–38.21) were associated with increased risk of unfavorable outcome. In a multivariate analysis, LOC (aOR 214.67, 95% CI 17.62–2615.89) and Fisher grade 4 (aOR 23.32, 95% CI 1.40–387.98) were significantly associated with poor functional outcome. WFNS grade of 4–5 was not included in the multivariate analysis to avoid multicollinearity (65% of patients with WFNS grade of 4 to 5 had LOC and 68% of patients with LOC had WFNS grade of 4 to 5).

**Table 4:** Univariate and multivariate logistic regression analysis on the risk factors for unfavorable outcome. WFNS grade of 4 to 5 and the need for ventriculostomy were not included in the multivariate analysis to avoid collinearity with LOC and hydrocephalus. Fisher grades 1–3 were used as reference value

	OR and its 95% CI	*P* value	aOR and its 95% CI	*P* value
Age	1.04 (0.99–1.08)	0.120	1.09 (1.00–1.18)	0.053
Female gender	1.23 (0.44–3.45)	0.688	1.67 (0.23–12.19)	0.612
Loss of consciousness	137.22 (30.76–612.21)	< 0.001	214.23 (17.58–2610.61)	< 0.001
WFNS 4–5	267.40 (47.54–1504.23)	< 0.001	–	
Radiological vasospasm	2.58 (0.88–7.54)	0.084	4.05 (0.54–30.42)	0.174
Fisher grade 4	21.00 (4.61–95.68)	< 0.001	23.24 (1.39–387.56)	0.028
Hydrocephalus	22.57 (6.71–75.85)	< 0.001	2.89 (0.40–20.91)	0.293
Ventriculostomy	12.71 (4.23–38.21)	< 0.001	–	
Shunt dependency	1.80 (0.21–15.61)	0.594	–	

## Discussion

We studied the clinical course and outcomes in 214 non-aneurysmal SAH patients, which is one of the largest cohorts of non-aneurysmal SAHs. Out of the 214 non-aneurysmal SAHs, we found that 2 patients had a re-bleed, totaling a re-bleed rate of 0.9%. Ninety-two percent of the patients had a favorable outcome. The patients with a NPSAH type of bleeding pattern had more frequently unfavorable outcome compared to patients with PSAH (16% vs 3%). In a multivariate analysis, the predictors for poor outcome were the LOC and the need for ventriculostomy, i.e., hydrocephalus on admission.

### Loss of consciousness as a strong predictor of poor outcome after non-aneurysmal SAH

In the multivariable logistic regression analysis, LOC was by far the most significant indicator of unfavorable outcome (aOR 137.94, 95% CI 30.92–615.41), regardless of the other factors. In addition, the positive predictive value for unfavorable outcome was 68% for all non-aneurysmal SAHs and 86% for NPSAHs, making LOC a clear clinical indicator of high risk for poor outcome. The significance of LOC could be explained by the fact that LOC is thought to be the result of early brain injury [18]. During a severe SAH, the leakage of blood causes a momentary increase in ICP and as the ICP rises, the cerebral perfusion pressure of the intracranial circulation may be not enough to guarantee the oxygen supply to the brain tissue resulting in a transient global ischemia. The lack of LOC in patients with PSAH (2/77) supports the idea that the source of PSAH is from other than arterial source, for example of venous origin around the midbrain [4, 7], as the bleed from venous origin does not cause such a momentary increase in ICP. In aneurysmal SAH, LOC is known to be a marker for early brain injury, and it is associated with 2.8-fold increase in the risk of unfavorable outcome [18]. Based on previous literature in aneurysmal SAHs [18, 19, 22] and our findings, it seems that regardless of the etiology of the bleed, LOC at onset is associated with early brain injury and furthermore with unfavorable outcome.

Since the level of consciousness is a major determinant for the WFNS grade, as well as for the other grading systems used to predict the outcome of SAH patients, LOC during ictus was highly associated with a high WFNS grade. We chose to focus on LOC rather than WFNS grade, since it seemed more sensitive than WFNS grading. Even though WFNS score is known to predict unfavorable outcome [17], some patients with a good WFNS score can have a poor outcome. The added value of using LOC as a clinical factor when estimating the risk of complications and poor outcome after a non-aneurysmal SAH in comparison to using traditional parameters such as Hunt and Hess, GCS, or WFNS grading is that presence and length of LOC can be used to stratify those patients who present in a good condition on admission (i.e., high GCS or good WFNS score) into those who nevertheless have high risk and those who do not. It is especially the patients who present with a good WFNS score (WFNS 1 to 3) for which additional risk stratification is needed (Table 1).

### Perimesencephalic vs non-perimesencephalic bleeding patterns

We found that the great majority of patients with unfavorable outcome had a NPSAH type of bleeding pattern (88%, 14/16). A recent study suggests that patients with PSAH have good prognosis, whereas patients with NPSAH are thought to have intermediate risk for complications that are often present in patients with aneurysmal SAH [2]. Our results support the idea that NPSAHs have intermediate clinical course: we found that 30% of patients with NPSAH developed hydrocephalus and 22% need ventriculostomy, which are higher rates reported to be associated with PSAH but lower than aneurysmal SAHs [3]. Although patients with NPSAH experience more often complications, still relatively large proportion of patients have a favorable prognosis (Table 2).

### Risk of vasospasm after non-aneurysmal hemorrhage is not negligible

Traditionally, it has been thought that non-aneurysmal SAHs are not associated with a significant risk of vasospasm. As in the aneurysmal SAHs [6], it is known that the greater amount of blood (Fisher 3 and 4) is associated with increased risk for vasospasm [11]. Previous studies have found that non-aneurysmal SAHs are associated with a 16–21% [1, 11, 12] risk of vasospasm. A similar risk for vasospasm was observed in our data (20%). For Fisher grades 1 and 2, vasospasm was seen in 8% (3/37) and 20% (1/5) of patients, respectively. However, 24% (26/108) of Fisher grade 3 and 20% (13/64) of Fisher grade 4 bleeding pattern patients had radiological vasospasm. Our results support the idea that the amount of leakage and the severity of the leakage are important for the development of vasospasm [13]. Vasospasm was more often seen in patients with LOC (32%) and in patients with Fisher 3 or 4 bleeding patterns compared to Fisher 1 and 2 bleeding patterns (23% vs 10%). Therefore, it seems that especially Fisher 3 and 4 graded non-aneurysmal SAHs should be treated according to the same protocol as the patients with aneurysmal SAHs to avoid vasospasm-induced complications.

### Hydrocephaly after non-aneurysmal hemorrhage

In our study, we found that risk for hydrocephalus was 17% and the risk for shunting 4%. Previous literature has suggested similar risk for hydrocephalus with rates varying from 16 to 30% [1, 9, 10]. However, the rate of shunting was substantially lower compared to previous findings by Schuss et al. (14%) [16] and Achrén et al. (17%) [1]. The difference between shunt rate of our cohort and the shunt rate of Achrén et al. was statistically significant (4% vs 17%, *P* =  < 0.001). It was an interesting finding, as the study setting by Achrén et al. in Finland is very similar to ours. The difference may be due to the fact that our study data also includes patients who were discharged from hospital with good neurological condition without structured follow-up and our study cohort included more patients with Fisher grade 1 bleeding pattern. In the study by Achrén et al., only patients with follow-up were included. We chose to include this patient group to avoid overestimating the risk of non-aneurysmal SAH-related complications.

### Strengths and limitations of the study

A strength of this study is the fact that, in Finland, all neurosurgical care of SAHs is centered in university hospitals, of which catchment areas are defined by the Finnish Government. Thus, all non-aneurysmal SAHs of our catchment area are first presented to TAUH making our study cohort comprehensive.

This study has some limitations that need to be addressed. Firstly, all patients did not have routine follow-up (39% of patients). This may introduce bias by underestimating the complications that manifest late after the onset of non-aneurysmal SAH. In a previous long-term follow-up study conducted at TAUH, however, no delayed mortality was observed as a result of non-aneurysmal SAH [14]. In addition, these patients had good neurological condition at the end of hospital stay (GOS 5), and thus, the clinical decision was that routine follow-up is not needed. Moreover, these patients are instructed to seek medical care if they have complications that manifest after discharge from hospital. Thus, it is reasonable to expect that these patients would have sought care from TAUH if they would have developed symptoms. Nonetheless, especially mild cognitive deficits or other neuropsychological symptoms might pass undiagnosed since routine neuropsychological testing after non-aneurysmal SAH has not been the clinical practice. Considering that the severity of the bleed appears to have a greater impact on the outcome of SAH, rather than the etiology of the bleed, we suggest that routine neuropsychological testing should be done for non-aneurysmal SAH patients in the future, especially for patients who present with more sever clinical course similar to aneurysmal SAH.

Secondly, patients with mild non-aneurysmal SAHs may never seek medical care, and thus, our reported rates of complications may overestimate the risk of non-aneurysmal SAH. Nevertheless, our data demonstrate that at least in more severe non-aneurysmal SAH serious complications may occur.

Third, in spite of our cohort of non-aneurysmal SAH patients being among the largest ones published, the number of serious end events was relatively low. This predisposes to unreasonably high ORs and confidence intervals in the multivariate models. While the ORs should be interpreted with caution, they do nevertheless clearly demonstrate that LOC, NPSAH, and Fisher 4 grade bleed are risk factors for poor outcome after non-aneurysmal SAH.

## Conclusions

As studied previously, most non-aneurysmal SAHs seem to have a benign clinical course, with little risk of re-bleed (0.9%). However, 8% of non-aneurysmal SAHs result in an unfavorable outcome, most of them being NPSAHs (88%). Bleeding pattern resembling aneurysmal SAH and LOC during ictus are factors that characterize patients with unfavorable outcome after non-aneurysmal SAH. Thus, while non-aneurysmal SAH is benign for most patients, it is important to provide intensive neurosurgical monitoring to patients who have a NPSAH bleeding pattern or have lost consciousness during the bleed.

### Supplementary Information

Below is the link to the electronic supplementary material.

Supplementary file1 (DOCX 35 KB)

## Data Availability

In accordance with the European Union’s GDPR regulations, the data used in this study cannot be made freely available. Pseudonymized dataset can, however, be shared after formal approval of a scientific research plan and data management plan by the local ethical review board and Tampere University Hospital.

## Abbreviations

TAUH: Tampere University Hospital
SAH: Subarachnoid hemorrhage
