# Correction to: The clinical course and outcomes of non-aneurysmal subarachnoid hemorrhages in a single-center retrospective study

**DOI:** 10.1007/s00701-023-05834-w

**Published:** 2023-10-25

**Authors:** Jeremias Tarkiainen, Valtteri Hovi, Liisa Pyysalo, Antti Ronkainen, Juhana Frösen

**Affiliations:** 1 Department of Neurosurgery, Tampere University Hospital and University of Tampere, Tampere, Finland; 2 Hemorrhagic Brain Pathology Research Group, Faculty of Medical Technology and Health Sciences, Tampere University, Tampere, Finland; 3 Department of Rehabilitation, Tampere University Hospital, Tampere, Finland

**Correction to: Acta Neurochirurgica a (2023) 165:2843–2853**

10.1007/s00701-023-05767-4

Unfortunately, we have just noticed a couple of errors in the numbers reported in our recently published manuscript “The clinical course and outcomes of non-aneurysmal subarachnoid hemorrhages in a single-center retrospective study”.

These errors are as follows:

1. In the Results section of abstract, the aOR and 95% CI for “loss of consciousness (LOC)” should be 214.23 (17.58–2610.61) instead of 214.67, 95% CI 17.62–2615.89.
2. In the Results section, subheading “Logistic regression analysis”, lines 9–10, the aOR and 95% CI for “loss of consciousness (LOC)” should be 214.23 (17.58–2610.61) instead of 214.67, 95% CI 17.62–2615.89.
3. In the Results section of abstract, the aOR and 95% CI for “Fisher grade 4 bleeding pattern” should be 23.24 (1.39–387.56) instead of 23.32, 95% CI 1.40–387.98
4. In the Results section, subheading “Logistic regression analysis”, lines 10–11, the aOR and 95% CI for “Fisher grade 4 bleeding pattern” should be 23.24 (1.39–387.56) instead of 23.32, 95% CI 1.40–387.98.
5. In the Discussion section, in page 8, paragraph 2, in the sentence “…In the multivariable logistic regression analysis, LOC was by far the most significant indicator of unfavorable outcome (aOR 137.94, 95% CI 30.92–615.41), regardless of the other factors.”. The aOR should have been 214.23, 95% CI 17.58–2610.61.
6. In the discussion section, page 8, paragraph 1, lines 9–11, the predictors of poor outcome should have been: “In a multivariate analysis, the predictors for poor outcome were the LOC and Fisher 4 grade bleeding pattern.”
7. In the Fig. 2, there was a small typo: The number in the “Number of patients with only one hospitalization related to aneurysm treatment: 500” should have been 510 instead of 500. This could be corrected in the Figure legend: “Number of patients with only one hospitalization related to aneurysm treatment should have been 510 instead of 500.”

We sincerely apologize for these mistakes having been missed during the check of page proofs.

